# Evaluating Predictive Pharmacogenetic Signatures of Adverse Events in Colorectal Cancer Patients Treated with Fluoropyrimidines

**DOI:** 10.1371/journal.pone.0078053

**Published:** 2013-10-22

**Authors:** Barbara A. Jennings, Yoon K. Loke, Jane Skinner, Melanie Keane, Gavin S. Chu, Richard Turner, Daniel Epurescu, Ann Barrett, Gavin Willis

**Affiliations:** 1 Norwich Medical School, University of East Anglia, Norwich, United Kingdom; 2 Norfolk and Norwich University Hospital, Norwich, United Kingdom; West German Cancer Center, Germany

## Abstract

The potential clinical utility of genetic markers associated with response to fluoropyrimidine treatment in colorectal cancer patients remains controversial despite extensive study. Our aim was to test the clinical validity of both novel and previously identified markers of adverse events in a broad clinical setting. We have conducted an observational pharmacogenetic study of early adverse events in a cohort study of 254 colorectal cancer patients treated with 5-fluorouracil or capecitabine. Sixteen variants of nine key folate (pharmacodynamic) and drug metabolising (pharmacokinetic) enzymes have been analysed as individual markers and/or signatures of markers. We found a significant association between *TYMP* S471L (rs11479) and early dose modifications and/or severe adverse events (adjusted OR = 2.02 [1.03; 4.00], p = 0.042, adjusted OR = 2.70 [1.23; 5.92], p = 0.01 respectively). There was also a significant association between these phenotypes and a signature of *DPYD* mutations (Adjusted OR = 3.96 [1.17; 13.33], p = 0.03, adjusted OR = 6.76 [1.99; 22.96], p = 0.002 respectively). We did not identify any significant associations between the individual candidate pharmacodynamic markers and toxicity. If a predictive test for early adverse events analysed the *TYMP* and *DPYD* variants as a signature, the sensitivity would be 45.5 %, with a positive predictive value of just 33.9 % and thus poor clinical validity. Most studies to date have been under-powered to consider multiple pharmacokinetic and pharmacodynamic variants simultaneously but this and similar individualised data sets could be pooled in meta-analyses to resolve uncertainties about the potential clinical utility of these markers.

## Introduction

Folate-dependent one-carbon metabolism is a target for drug groups that are widely used in the treatment of cancer and inflammatory diseases. Two of the drugs, 5-fluorouracil (5-FU) and capecitabine, are central to the medical management of colorectal cancer in both advanced and adjuvant settings; they are used as monotherapies or in combination with other chemotherapeutic and biological agents. Both folate metabolism and the catabolism of 5-FU and capecitabine depend on a number of enzymes that are functionally polymorphic [1]. 5-FU is a fluoropyrimidine that has been used as a chemotherapeutic agent for more than five decades. Inhibition of thymidylate synthase (TYMS) is an important mechanism of action for 5-FU, which leads to inhibition of DNA synthesis and DNA repair. This cytotoxicity is partly dependent on the formation of a ternary complex between TYMS, the deoxyribonucleoside derived from 5-FU and 5-methyl-tetrahydrofolate (THF). This can be enhanced by the expansion of reduced folate pools, which can be achieved pharmacologically because 5-FU regimes include folinic acid (Leucovorin), a 5-formyl derivative of THF [2]. 

The fluoropyrimidine, capecitabine (a pro-drug that is preferentially converted to 5-FU in tumour cells), has been designed for oral administration and to be more specific than 5-FU, thus leading to potential differences in the safety profile [2-5].

The use of 5-FU/leucovorin in randomized controlled trials of adjuvant chemotherapy for colorectal cancer has been shown to improve both disease-free and overall survival as compared to surgery alone [6,7]. Subsequently, trial participants receiving 5-FU/leucovorin combined with oxaliplatin were found to have significantly improved progression-free survival of 9.0 months compared with 6.2 months in those receiving 5-FU/leucovorin alone (p<0.001) [8]. However, neutropenia and diarrhoea were important adverse effects noted in this trial. The need to manage toxicity is important because unintended effects may result in the patient having to receive a lower dose or shorter course of chemotherapy, with adverse consequences on the benefit/harm balance. Hence, there are potential clinical advantages from the development of predictive markers to guide clinicians in selecting individuals who are most likely to benefit (or least likely to be harmed) from a particular drug regimen. If individuals with high susceptibility for adverse effects could be identified before treatment, strategies to reduce the risk such as using alternative chemotherapy regimens (based on different agents or dose modification) and closer monitoring with greater use of supportive therapeutics, could be applied. Many studies have explored the predictive value of genotyping for beneficial response to chemotherapy and the likelihood of chemotherapy related adverse events [9-20] and heterogeneous conclusions were drawn about the association of individual markers with treatment outcomes. A recent genome-wide association study identified one variant that had not been previously implicated in 5-FU pharmacokinetic or pharmacodynamic pathways and failed to identify association signals in previously identified markers or their imputed proxys [21].

We have previously reported a meta-analysis on the clinical impact of *TYMS* and methylenetetrahydrofolate reductase (*MTHFR*) [22]. Data were synthesized from more than 2000 patients for the most commonly studied markers *TYMS* 5’ UTR repeat polymorphism (rs45445694) and *MTHFR* 677 C>T (rs1801133). We found a statistically significant association between clinical response and the *TYMS* genotype associated with low protein expression only; however, the effect size is small (RR = 1.36 [1.11, 1.65] and RR = 2.04 [1.42, 2.95] for benefit and adverse events respectively) and therefore suggests limited clinical utility for this marker. Some SNPs of the *DPYD* gene have been strongly associated with severe toxicity; an exon-skipping mutation in intron 14 (rs3918290) has been shown to have a positive predictive value ranging from 46 % [23] to 100 % [17,24]. The benefit/harm ratio is likely to depend on a complex polygenic model where individual genotypes have only a small role. Analysis of multiple polymorphisms simultaneously will allow us to consider additive, synergistic and compensating variants of folate metabolism and anti-folate catabolism that may have clinical utility as predictive genetic signatures; but data from large cohorts will be needed. In this study we present individualised pharmacogenetic patient data that could potentially be pooled in meta-analyses of gene interactions. Our objectives were to test the clinical validity of previously identified markers of adverse events in a broad clinical setting; and to identify any novel associations between adverse events and candidate variants of proximal enzymes in the pharmacodynamic and pharmacokinetic pathways.

## Materials and Methods

### Study Design

This is an observational pharmacogenetic cohort study of colorectal cancer patients treated with 5-FU or capecitabine. 

### Participants, Setting and Treatment Regimens

Peripheral blood samples were collected from two hundred and fifty-four CRC patients treated within the Oncology department of the Norfolk and Norwich University Hospital in Norfolk, England between 2008 and 2011. The Central Office for Research Ethics Committees approved the study protocol (REC reference 07/H0310/134) and written informed consent was obtained from all participants.

The patients, who had a World Health Organisation performance status of between 0 and 2, were treated in neo-adjuvant, adjuvant and palliative settings with either intravenous 5-FU or capecitabine as monotherapy, or combined with other agents as dual therapy. The second chemotherapy agents were typically irinotecan (FOLFIRI, CAPIRI regimens) or the platinum salt oxaliplatin (FOLFOX, CAPOX regimens) [25]. Toxicities encountered with all agents used were assessed according to the Common Terminology Criteria for Adverse Events (CTCAE) Version 4.0. The standard departmental protocol was followed for dose modification and treatment withdrawal.

### Baseline Characteristics

The age, sex, laboratory data for bone marrow and liver function, plasma levels of carcinoembryonic antigen (CEA) and disease stage were recorded for each participant at the start of the treatment regime. The histological classification and grade of the primary tumour were also recorded.

### Phenotypes

Relevant clinical data about adverse events were collected from patient records and laboratory charts for 12 weeks from the start of the treatment regime. Adverse events were graded in accordance with the CTCAE version 4.0 for gastrointestinal symptoms, mucositis/stomatitis, palmar-plantar syndrome, paraesthesia and cardiac toxicity, neutropenia, anaemia, thrombocytopenia and abnormal liver function tests.

1. Any delays or reductions in the administration of fluoropyrimidines due to adverse events were recorded as primary outcomes for the subsequent analyses.
2. Grade 3, 4 or 5 adverse events were analysed as secondary outcomes. Toxicity classified as paraesthesia was not included in the statistical analysis of patients treated with combination chemotherapies because the symptom is largely attributable to oxaliplatin therapy. Abnormal liver function tests were not included in the analyses for participants with liver metastases.

### Genes, Genetic Variants and Genotype Analysis and Analytic Validity

We used or developed allelic discrimination assays for the polymorphic forms of *MTHFR*, *TYMS*, *DHFR*, *MTHFD*, *SHMT, DPYD, UMPS, CDA and TYMP* described in Table 1. We identified the genetic variants of interest through the systematic extraction of data for polymorphisms of the genes described on the *NCBI* and *SNP 500* databases or in publications, and their stratification was based on published data or sequence-based predictions about their functional impact [17,18,26-34]. 

**Table 1:** The genetic markers analysed as predictive markers of adverse events and biochemical response to 5FU and Capecitabine treatment.

**1 (a**)	**Gene symbol, locus**	**NCBI SNP I.D; Polymorphism; class of mutation**	**PCR primer pair; and Taqman probes**	**Enzyme used for RFLP analysis**	**1 (b**)** Genotype Frequency homozygotes for major allele/ heterozygotes/ homozygotes for minor allele**
**Markers of Pharmacodynamics**	*TYMS*, 18p11.32	rs45445694; 5’UTR; Tandem repeat polymorphism (2R/3R)	AAAAGGCGCGCGGAAG and GCCGGCCACAGGCAT	Not applicable, gel analysis	84/116/53
		G>C in 3R alleles of rs45445694; SNP	AAAAGGCGCGCGGAAG and GCCGGCCACAGGCAT	Hae III	147/89/17
		rs16430; 3’UTR 1494 -6bp/+6bp; In/del	GCAGAACACTTCTTTATTATAGCAACATATAA and CGATCATGATGTAGAGTGTGGTTATG	Not applicable, gel analysis	124/103/26
	*MTHFR*, 1p36.3	rs1801133; 677C>T; A222V, Missense	GGGTCAGAAGCATATCAGTCATG and CACAAAGCGGAAGAATGTGTC	HinfI	110/111/32
		rs1801131; 1298A>C; E429A, Missense	CTACCTGG*AGAGCAAGTCCCCCAA and GGATGAACCAGGGTCCCC	MboII	117/119/17
	*DHFR*, 5q14.1	IVS1+59_60insACCTGGGCGGGACGCGCCA; 19 bp intron 1 in/del	ATGGGACCCAAACGGGC and CACCCTTCCTGCCAGCG	Not applicable, gel analysis	62/135/56
	*MTHFD1*, 14q24	rs2236225; 1958G>A; R653Q, Missense	TTCCAATGTCTGCTCCAAATCC and CCTTCCGATTCCAAATCAATTC	Msp1	81/123/49
	*SHMT1*, 17p11.2	rs1979277; 1420C>T; L474F, Missense	GCCCGCTCCTTTAGAAGTCA and CTCCGGGAGGAGGTTGAGA; VIC TTCGCCTCTTTCTTC and FAM TTCGCCTCTCTCTTC	Not applicable, Taqman probes	125/107/21
**Markers of Pharmacokinetics**	*DPYD*, 1p22	rs3918290; IVS14+1 G>A; Exon-skipping SNP in intron 14	CCTCTGGCCCCATGTATG and AGCAACTGGCAGATTCTTTAATAAA	HpyCH4IV	250/3/0
		1236G>A; E412E, Synonymous SNP	CTATGCAGTTTGTTCGGACT*GA and GATGACCACATCGGCTTTCA	DdeI	243/10/0
		rs67376798; 2846A>T; D949V, Missense	TAGAGCAAGTTGTGGCTATGATC*G and GTCTCATAGCATTCTAATTCCAGCA	TaqαI	251/2/0
		c1129-5923C>G; intronic SNP creates splice site	TTTTATTTCACTCG*GCATCAGCC and CATTTGACAAATCAGGTTGTCACTT	DdeI	243/10/0
	*UMPS*,3q13	rs1801019; 638G>C; G213A, Missense	TGTGGCAGCGAATCATAC*TG and GGATCCTGGGCAGCTCT	BsrI	174/73/6
	*CDA*, 1p36	rs2072671; 258A>C; K27Q, Missense	GCTCCCA GGAGGT*CAAG and TTACCTTTGAAGATTCTCCCCT	Hpy188III	113/110/30
	*TYMP*, 22q13	rs11479; 1412C>T; S471L, Missense	GCAGGAGGCGCTCGT and CTGACAAGGTTTCGCGGC	MnlI	207/44/2
		rs112723255; 1393G>A; A465T, Missense	GCAGGAGGCGCTCGT and CTGACAAGGTTTCGCGGC	HinP1I	234/17/2

**1 (a**) The table describes the functional impact of each polymorphism and the oligonucleotides and restriction enzymes used in the assays.

**1 (b**) The distribution of genotypes for each marker is presented for the cohort of 253 colorectal cancer patients included in the pharmacogenetic analysis.

(* - indicates a mismatch with the wild type sequence; introduced to eliminate/create enzyme sites for the assay)

DNA was extracted from whole blood using standard methods and sub-aliquoted onto 96 well plates at a concentration of approximately 100 ng µl^-1^. All subsequent reactions were also performed in 96 well plates and 8 channel automatic pipettes were used for all liquid transfers. Fifteen of the PCR reactions comprised 100 ng DNA, 200 nmol L^-1^ of each primer and 1 x PCR Thermo Start Mastermix (ABgene UK, Epsom, England) in a 25 µl volume. The PCR conditions for each assay varied according to cycle number and annealing temperature but in each case an initial denaturation was performed at 95 °C for 5 minutes and the PCR reaction was linked to a final extension step of 10 minutes at 70 °C.

Restriction fragment length polymorphism (RFLP) analysis was used for 12 of the assays. In each case, 10 µl of PCR product was digested overnight at 37 °C in a 20 µl reaction volume. The enzymes used (New England Biolabs, Hitchin, UK) for each reaction are described in Table 1(a). The PCR products were electrophoresed on a 1 X Tris/borate/EDTA, 3% Metaphor agarose (FMC Bioproducts, Lichfield, UK) gel in a Stretch-wide apparatus (ABgene, Epsom, UK) at 80 V for 50 minutes. 

For the *SHMT1* assay ‘an Assay by Design’ kit of primers and Taqman probes were used with Taqman mastermix (Applied Biosystems, Warrington, UK) and 100 ng of DNA in a 25µl volume. The Applied Biosystems standard minor groove binding PCR reaction conditions were used; 50 °C for 2 minutes; 95 °C for 10 minute followed by 40 cycles of 60 °C for 1 minute and 92 °C for 15 seconds. 

A number of control steps were included in our standard operating procedures.

1. Genotype-specific and no DNA controls were used.
2. A minimum of 10% of the samples from each batch were genotyped a second time.
3. The 677C>T and 1298A>C alleles of the *MTHFR* loci have been shown to be in linkage disequilibrium because these variants are very rarely found in *cis* [33,35,36]. The c1129-5923C>G intronic SNP of *DPYD* is tightly linked to 1236G>A, and therefore these SNPs are expected to be found as a diplotype.
4. We used the exact test of Hardy-Weinberg proportions to analyse the frequencies of the genotypes detected for each locus for deviation from Hardy-Weinberg equilibrium (HWE).

### Statistical Analysis

The main aim of the study was to assess the association between individual candidate SNPs and toxicity. The adjusted test for trend was pre-specified as our method for statistical analysis but for completeness, and to make our data available for meta-analysis by others, we also calculated associations using dominant and recessive models.

For unadjusted results, we calculated odds ratios (ORs) and 95% confidence intervals (CIs) directly between the risk of both 5-FU dose modification and the risk of grade 3, 4 or 5 toxicity events and each SNP. We took homozygotes for the major allele as the reference category. We used the score test for trend of odds. For adjusted results, we used unconditional logistic regression to estimate the ORs and 95% CIs between risk of the two outcomes and each SNP, adjusted for age, sex, previous chemotherapy and treatment regime. 

To calculate the adjusted test for trend, we fitted the SNP result (0, 1 or 2) as a continuous outcome. For completeness, we also calculated whether alleles increased disease risk under dominant (1+2 versus 0) and recessive (2 versus 0+1) models. Where there were small cell counts (expected value < 5) we used Fisher’s exact estimate for the unadjusted results and we did not calculate the results for the adjusted model. We analysed the data using Stata version 12 (StataCorp, 2011). Many tests were carried out so, whilst not formally adjusting for multiple comparisons, we pre-specified that the results of the adjusted test for trend should be taken as the main results. 

## Results

### Participants and treatment regimens

254 participants were recruited to the study. One participant was subsequently excluded who was undergoing combination chemo-radiotherapy for a squamous cell carcinoma of the anus. 

Patient characteristics are summarised in Table 2. The median age of the participants was 67 years and the male to female ratio was 1.34: 1. Most participants (209, 82.60 %) were undergoing first-line chemotherapy. 

**Table 2:** Demographic, clinical and pathological information for 253 colorectal cancer patients.

Characteristic		Number (%)
Males		145 (57.31)
Females		108 (42.68)
Median age		67
Age range		23 - 88
Histology	Adenocarcinoma	221 (87.01)
	Mucinous adenocarcinoma	25 (9.84)
	Other / Unknown	7 (2.76)
Modified Dukes Classification at diagnosis*	A	6 (2.36)
	B	56 (22.05)
	C1	121 (47.64)
	C2	26 (10.24)
First line Chemotherapy	Yes	209 (82.60)
	No	44 (17.39)
Treatment regime	5-FU monotherapy	63 (24.90)
	Combination chemotherapy with 5-FU	31 (12.25)
	Capecitabine monotherapy	58 (22.92)
	Combination chemotherapy with capecitabine	101 (39.92)

* Dukes score from post-surgical histopathology reports where available.

### Phenotype

109 of the 253 (43.08 %) participants included in the pharmacogenetic analysis had a dose delay or modification, and 44 (17.39 %) had a grade 3 or 4 adverse event; or died from causes (gastrointestinal symptoms, liver failure, cardiac symptoms and thromboembolic disease) that were considered unrelated to disease progression within 12 weeks of commencing their chemotherapy regime. Severe adverse events were observed more frequently for patients receiving combination chemotherapy in comparison with those receiving fluoropyrimidine monotherapy (22 % versus 12 % respectively; see Table S1). 

### Genotype Analysis and Analytic Validity

Scoring of Genotypes was 99.5 % successful in the first round of assays and 100 % successful after any failed samples and repeats had been re-assayed.

The genotype frequencies are described in Table 1 (b). The most common minor allele (*DHFR*) had a frequency of 49 % and the rarest minor allele (*DPYD*, rs67376798) had a frequency of 0.4 %. These frequencies concurred with those found on databases for other northern European populations which is in keeping with our previous observation that 94 % of the population served by the recruiting hospital are white and born in England [37].

There were no inconsistencies in the data analysis of the control markers included with each batch, or with duplicate analyses, or with observations about linkage disequilibrium. No significant deviations from HWE were observed.

### Statistical Analysis

The relationships between each SNP and dose modifications or severe toxicity are presented in Table S2. Odds ratios (ORs) adjusted for age, sex, previous chemotherapy and treatment regime are presented. For *MTHFR* and *DPYD* SNPs, we have included an analysis of variants with the same functional effects as signatures of alternative polymorphisms. 

The results of the adjusted test for trend were pre-defined as our main hypothesis-testing data. But for four loci there were zero observations of homozygotes for the minor allele, in which case we used the adjusted dominant model. 

We found a significant association between early dose modifications and severe adverse events (adjusted OR = 2.02 [1.03; 4.00], p = 0.042, adjusted OR = 2.70 [1.23; 5.92], p = 0.013 respectively from the dominant model) and *TYMP* (rs11479). There was also a significant association between these phenotypes and a signature of *DPYD* mutations (Adjusted OR = 3.96 [1.17; 13.33], p = 0.03, adjusted OR = 6.76 [1.99; 22.96], p = 0.002 respectively). We also found a significant trend for *TYMP* (rs11479) based on two observed homozygotes for the minor allele.

Adjusted results that we do not emphasise (to avoid multiple comparisons and because a significant test for trend is the best signal of an effect) were (1) an association for dose modification and *DHFR* in/del heterozygotes; adjusted OR of 2.19 [1.12; 4.28], p=0.023, and OR of 2.15 [1.13; 4.08], p=0.020 in the dominant model (2). An association for severe toxicity and or *SHMT1* (rs1979277) heterozygotes; an adjusted OR of 0.40 [0.18; 0.88], p=0.023 compared to CC homozygotes, and OR of 0.47 [0.23-0.97], p=0.041 in the dominant model. No other adjusted results were significant.

### Clinical Validity and Impact

Table S1 shows individualised data for the markers associated with severe adverse events (*DPYD* and *TYMP* genotypes) and clinical phenotype data for the 44 participants who had severe adverse events. Nineteen of the 44 participants with severe adverse events carried at least one of the candidate predictive markers. 

If detection of the combined SNP signature was used as a diagnostic procedure to identify those who would subsequently suffer severe adverse events, the sensitivity would be 45.5 %, with a positive predictive value of 33.9 %. The potential impact of testing for the combined SNP signature and then changing to a different chemotherapy regimen in affected individuals can be estimated in a hypothetical clinical population of 1000 patients. If those patients had similar characteristics to those within our cohort, 233 of them would be combined SNP signature positive. The impact of changing the regimen in these 233 patients would be 79 fewer severe adverse events. Here, 95 patients would still have severe adverse events (down from the original 174 with no testing performed). However, 156 patients with the combined SNP signature would have had their regimen changed for no specific benefit because they would not have gone on to develop severe adverse events.

## Discussion

We present an analysis of functionally important genetic variants in the pharmacokinetic and pharmacodynamic pathways that influence response to fluoropyrimidines. The phenotypes examined were adverse events that were identified by a dose delay or dose modification within 12 weeks due to toxicity, and by CTCAE grade 3, 4 or 5 scores. 

### Pharmacokinetics

Each of the pharmacokinetic variants that we consider are compelling candidates as predictive markers because they have a known or putative functional impact on the enzymes needed for drug catabolism or their metabolism to an active form.

The *DPYD* variants analysed lead to enzyme deficiency or absence and their functional effects can be observed in heterozygous carriers. However, each *DPYD* SNP is rare, which reduces their potential clinical utility as predictive markers. In this study therefore we have also considered these *DPYD* variants as a signature of alternative polymorphisms, and found a strong association with early adverse events (see Tables S1 and S2). 

*TYMP* encodes thymidine phosphorylase; the activity and expression of which has a reported role in tumorigenesis as well as activation of 5-FU and capecitabine [38]. We present a novel finding about a variant that should now be tested in an independent cohort. There is a significant association between early adverse events and the *TYMP* SNP rs11479 (see tables S1 and S2), the minor allele results in an amino acid substitution (e.g. NP_001107227.1:p.Ser471Leu, though alternate splice forms have been described). The importance of this particular variant is unclear; the amino acid substitution occurs just outside the pyrimidine nucleoside phosphorylase C terminal domain in most models and the Ser at this position is not widely conserved in mammals. However, the variant could also be in linkage disequilibrium with another polymorphism that is functionally important. One previous pharmacogenetic study of *TYMP* SNPs, including rs11479, failed to find an association with the adverse event palmar-plantar syndrome in a small (n = 130) mixed cohort of breast and colorectal cancer patients treated with capecitabine [39]. In another small study of colorectal cancer patients (n = 60) no association with survival was found for a synonymous *TYMP* SNP, rs470119 [40]. 

We found no significant associations or trends for early dose modifications or severe adverse events with the candidate variants of *UMPS* (rs1801019) and *CDA* (rs2072671). Genetic variants for these loci have previously been associated with severe neutropenia and diarrhoea in patients treated with 5-FU [41], and with palmar-plantar syndrome in patients treated with capecitabine [39].

### Pharmacodynamics

Inhibition of thimydylate synthase is an important pharmacodynamic mechanism for fluoropyrimidines but the balance of folate species within the biochemical pathway may depend on the enzyme variants at key branch points [1]. We have therefore examined candidate polymorphisms for *MTHFR*, *DHFR*, *MTHFD1*, and *SHMT* in addition to *TYMS*. These are non-synonymous SNPs or variants that affect untranslated control regions; each polymorphism has a demonstrated or putative influence on gene expression or function. 

No significant associations or trends were found for individual polymorphisms that have been classified as low activity thymidylate synthase variants and toxicity within 12 weeks (Table S2). In the analysis of *TYMS* genotypes, it has been suggested that haplotype rather than genotype analysis may improve the sensitivity and specificity of pharmacogenetic testing. The G>C polymorphism in nucleotide 12 of the TYMS 28 bp VNTR repeat elements has been proposed to affect both expression of TYMS *in vitro* and levels of 2’-deoxyuridine *in vivo*. However, the published data are inconclusive, the majority of the possible genotypes have not been examined in relation to 5-FU sensitivity; different methods have been used to test gene expression; and conclusions about the putative functional effects have not been congruent [28,42-45]. Another haplotype of clinical interest [46] comprises the 5’ and 3’ *TYMS* variants, rs45445694 and rs16430 (also referred to as rs34489327) for which there is linkage disequilibrium [9,12,32]; but again there have been conflicting findings about the functional impact of the 3’ polymorphism [19,32,47]. 

In our previous systematic review and recent literature search for pharmacogenetic studies of colorectal cancer patients treated with fluoropyrimidines, no other studies were identified that included an analysis of *DHFR*, *SHMT* or *MTHFD1* genotypes. Associations with an increased risk of dose modification were identified for particular *DHFR* genotypes and decreased risk of severe adverse events with particular *SHMT* genotypes in this study. But we do not emphasise these results because they derive from the adjusted dominant model only and there was no concordance between the 2 phenotypes considered (Table S2). 

In summary, we did not identify any significant or compelling associations between the individual candidate pharmacodynamic markers and toxicity. This may reflect the complexity of the intrinsic and extrinsic factors that affect fluoropyrimidine response including dietary folate; leucovorin provided as part of the therapeutic regime; and variability in folate uptake.

### Limitations of Observational Studies

The observational nature of this study means that potential bias cannot be excluded. There are a number of factors that could bias towards the null. This may stem from incomplete recording of adverse events, missing data, or patients having early dose modifications and/or prophylactic interventions before higher grade events occurred. Small effect sizes, coupled with low allele frequency in some instances would have reduced the power of the study to detect any significant association. Conversely, type I error may also occur in erroneously reporting a significant finding when there is actually no true association. This can stem from multiple testing of a diverse range of genetic markers (particularly with post-hoc or ‘data trawling’ analyses) and is a problem that may be ameliorated through a Bonferroni correction. However, Perneger highlights a number of methodological weaknesses with Bonferroni corrections (such as an increased risk of type II errors or false-negatives), and he recommends that Bonferroni correction ‘should not be used when assessing evidence about specific hypotheses’[48]. This point is particularly relevant to our study because we have focused on the evaluation of pre-specified variants selected through rigorous review of the literature, and we have only highlighted associations identified through adjusted tests for trend and *a priori* hypotheses. Equally, unmeasured or residual confounding may explain differences between groups, despite our statistical adjustments for known confounders such as treatment protocol; for example, the response to CAPOX, FOLFORI and FOLFOX protocols can also be influenced by variants of enzymes that are not part of fluoropyrimidine metabolism. However doctors, patients and researchers were all blinded to the genotype status of the patients throughout the study, thus making it less likely that patients with particular genotypes were selected, monitored or treated differently (selection, detection or performance biases being unlikely due to Mendelian randomization). 

### Conclusion and Future Directions

In conclusion, these data identify and confirm markers that predict toxicity but our analysis of their clinical validity indicates limited utility. This has important implications for helping clinicians and patients arrive at evidence-based decisions on the pros and cons of investing in commercially available genotyping tests for predicting 5-FU toxicity during treatment of colorectal cancer. 

Only a few pharmacogenetic studies of the genotypes described have considered the role of epistasis but some interactions have been identified that warrant further analysis [19,46,49,50]. Most studies to date have been under-powered to consider multiple pharmacokinetic and pharmacodynamic variants simultaneously but this (Table S3) and similar individualised data sets can be pooled in meta-analyses to resolve uncertainties about the potential clinical utility of these markers and their combined signatures.

## Supporting Information

Table S1 **The genotypes at the loci DPYD and TYMP for 44 participants who had grade 3, 4 or 5 adverse events within 12 weeks of starting the chemotherapeutic protocol.** Treatment regimes; 1 = 5-FU as monotherapy; 2 = 5FU in combination chemotherapy; 3 = capecitabine as monotherapy; 4 = capecitabine in combination chemotherapy.
For the genotype data; 0 = homozygous for the minor allele; 1 = heterozygous; 2 = homozygous for the major (wild type) allele. The genotypes 1236G>A and c1129-5923C>G are in linkage disequilibrium.
LFT; liver function tests.
(DOC)

Table S2 **Analyses of associations between fluoropyrimidine toxicity and genotype.** **The results from the test for trend and from dominant and recessive genetic models are shown**.
(a) Main effect of polymorphisms on fluoropyrimidine dose modification: markers of pharmacodynamics.
† Adjusted for age, sex, previous chemotherapy and treatment regime using logistic regression ‡Fisher’s exact estimate used *Test for trend could not be calculated because of 0 observations in one or both phenotype groups for homozygotes in the minor allele.
(b) Main effect of polymorphisms on fluoropyrimidine dose modification: markers of pharmacokinetics.
† Adjusted for age, sex, previous chemotherapy and treatment regime using logistic regression ‡Fisher’s exact estimate used.
(c) Main effect of polymorphisms on grade 3, 4 or 5 toxicity events: markers of pharmacodynamics.
† Adjusted for age, sex, previous chemotherapy and treatment regime using logistic regression ‡Fisher’s exact estimate used.
(d) Main effect of polymorphisms on grade 3, 4 or 5 toxicity events: markers of pharmacokinetics.
† Adjusted for age, sex, previous chemotherapy and treatment regime using logistic regression ‡Fisher’s exact estimate used *Test for trend could not be calculated because of 0 observations in one or both phenotype groups for homozygotes in the minor allele.
(DOCX)

Table S3 **Individualised data for each toxicity phenotype.**
(XLS)
