# Correction: Evaluating Predictive Pharmacogenetic Signatures of Adverse Events in Colorectal Cancer Patients Treated with Fluoropyrimidines

**DOI:** 10.1371/journal.pone.0124893

**Published:** 2015-04-13

**Authors:** Barbara A. Jennings, Yoon K. Loke, Jane Skinner, Melanie Keane, Gavin S. Chu, Richard Turner, Daniel Epurescu, Ann Barrett, Gavin Willis

There are errors in Table S1 of the published paper. Please view the correct Table S1 here.

There is an error in the Results. The Results should state that twenty of the 44 participants with severe adverse events carried at least one of the candidate predictive markers.

## Supporting Information

S1 Table The genotypes at the loci DPYD and TYMP for 44 participants who had grade 3, 4 or 5 adverse events within 12 weeks of starting the chemotherapeutic protocol. Treatment regimes; 1 = 5-FU as monotherapy; 2 = 5FU in combination chemotherapy; 3 = capecitabine as monotherapy; 4 = capecitabine in combination chemotherapy. For the genotype data; 0 = homozygous for the minor allele; 1 = heterozygous; 2 = homozygous for the major (wild type) allele. The genotypes 1236G>A and c1129-5923C>G are in linkage disequilibrium.
LFT; liver function tests.
(XLS)
