# Supplementary material for: Evaluating Predictive Pharmacogenetic Signatures of Adverse Events in Colorectal Cancer Patients Treated with Fluoropyrimidines
Source: PLoS One. 2013 Oct 22;8(10):e78053. doi: 10.1371/journal.pone.0078053 (PMC3805522; doi:10.1371/journal.pone.0078053)
Supplement: Table S1 — The genotypes at the loci DPYD and TYMP for 44 participants who had grade 3, 4 or 5 adverse events within 12 weeks of starting the chemotherapeutic protocol. Treatment regimes; 1 = 5-FU as monotherapy; 2 = 5FU in combination chemotherapy; 3 = capecitabine as monotherapy; 4 = capecitabine in combination chemotherapy. For the genotype data; 0 = homozygous for the minor allele; 1 = heterozygous; 2 = homozygous for the major (wild type) allele. The genotypes 1236G>A and c1129-5923C>G are in linkage disequilibrium. LFT; liver function tests. (DOC) [file pone.0078053.s001.doc]

| **Treatment regime** | **Sex** | **Age in years** | **Phenotype** | ***DPYD*** rs3918290 | ***DPYD***  1236G>A | ***DPYD*** rs67376798 | ***DPYD***  c1129-5923C>G | ***TYMP***  rs11479 |
| --- | --- | --- | --- | --- | --- | --- | --- | --- |
| 1 | F | 71 | Neutropenia | 2 | 2 | 2 | 2 | 1 |
| 1 | F | 68 | Neutropenia, Abnormal LFT | 1 | 2 | 2 | 2 | 2 |
| 1 | F | 63 | Gastrointestinal symptoms | 2 | 2 | 2 | 2 | 2 |
| 1 | F | 71 | Gastrointestinal symptoms | 2 | 2 | 2 | 2 | 1 |
| 1 | F | 76 | Gastrointestinal symptoms, Abnormal LFT | 2 | 2 | 2 | 2 | 2 |
| 1 | F | 76 | Cardiac toxicity | 2 | 2 | 2 | 2 | 2 |
| 2 | M | 55 | Neutropenia | 2 | 2 | 2 | 2 | 2 |
| 2 | F | 65 | Neutropenia | 2 | 2 | 2 | 2 | 1 |
| 2 | M | 57 | Neutropenia | 2 | 2 | 2 | 2 | 2 |
| 2 | F | 47 | Neutropenia | 2 | 2 | 2 | 2 | 2 |
| 2 | M | 58 | Neutropenia | 2 | 2 | 2 | 2 | 2 |
| 2 | F | 65 | Neutropenia, Gastrointestinal symptoms | 2 | 2 | 2 | 2 | 2 |
| 2 | F | 59 | Gastrointestinal symptoms | 2 | 2 | 2 | 2 | 1 |
| 2 | F | 69 | Gastrointestinal symptoms, Palmar plantar syndrome | 2 | 2 | 2 | 2 | 2 |
| 2 | F | 62 | Gastrointestinal symptoms, Abnormal LFT | 1 | 2 | 2 | 2 | 2 |
| 2 | M | 74 | Death from cardiac toxicity, Abnormal LFT | 2 | 2 | 2 | 2 | 2 |
| 2 | M | 68 | Death from liver failure, Abnormal LFT | 2 | 2 | 2 | 2 | 1 |
| 2 | M | 72 | Death from thromboembolic disease | 2 | 2 | 2 | 2 | 2 |
| 3 | F | 72 | Gastrointestinal symptoms | 2 | 2 | 2 | 2 | 1 |
| 3 | F | 68 | Gastrointestinal symptoms | 2 | 2 | 2 | 2 | 2 |
| 3 | F | 68 | Gastrointestinal symptoms | 2 | 2 | 1 | 2 | 2 |
| 3 | M | 77 | Gastrointestinal symptoms | 2 | 2 | 2 | 2 | 2 |
| 3 | M | 69 | Palmar plantar syndrome | 2 | 2 | 2 | 2 | 2 |
| 3 | F | 69 | Palmar plantar syndrome | 2 | 2 | 2 | 2 | 1 |
| 3 | M | 45 | Abnormal LFT (&Gilbert's syndrome) | 2 | 1 | 2 | 1 | 0 |
| 3 | F | 64 | Abnormal LFT | 2 | 2 | 2 | 2 | 2 |
| 3 | M | 70 | Cardiac toxicity | 2 | 2 | 2 | 2 | 2 |
| 4 | M | 69 | Neutropenia | 2 | 2 | 2 | 2 | 2 |
| 4 | M | 59 | Neutropenia | 2 | 2 | 2 | 2 | 2 |
| 4 | F | 59 | Neutropenia | 2 | 2 | 2 | 2 | 2 |
| 4 | M | 63 | Neutropenia, Gastrointestinal symptoms | 2 | 2 | 2 | 2 | 1 |
| 4 | F | 63 | Death from Gastrointestinal symptoms, Neutropenia | 2 | 2 | 1 | 2 | 2 |
| 4 | F | 61 | Gastrointestinal symptoms | 2 | 2 | 2 | 2 | 2 |
| 4 | F | 50 | Gastrointestinal symptoms | 2 | 2 | 2 | 2 | 2 |
| 4 | F | 63 | Gastrointestinal symptoms | 2 | 2 | 2 | 2 | 1 |
| 4 | M | 67 | Gastrointestinal symptoms | 2 | 1 | 2 | 1 | 2 |
| 4 | M | 66 | Gastrointestinal symptoms | 2 | 2 | 2 | 2 | 2 |
| 4 | F | 67 | Gastrointestinal symptoms | 2 | 2 | 2 | 2 | 2 |
| 4 | F | 77 | Gastrointestinal symptoms | 2 | 2 | 2 | 2 | 1 |
| 4 | F | 56 | Palmar plantar syndrome | 2 | 2 | 2 | 2 | 1 |
| 4 | M | 62 | Palmar plantar syndrome, Paraesthesia | 2 | 2 | 2 | 2 | 1 |
| 4 | M | 65 | Cardiac toxicity | 2 | 2 | 2 | 2 | 1 |
| 4 | F | 41 | Cardiac toxicity | 2 | 2 | 2 | 2 | 2 |
| 4 | M | 66 | Myocardial infarction, Abnormal LFT | 2 | 2 | 2 | 2 | 2 |
