# Supplementary material for: Evaluating Predictive Pharmacogenetic Signatures of Adverse Events in Colorectal Cancer Patients Treated with Fluoropyrimidines
Source: PLoS One. 2013 Oct 22;8(10):e78053. doi: 10.1371/journal.pone.0078053 (PMC3805522; doi:10.1371/journal.pone.0078053)
Supplement: Table S2 — Analyses of associations between fluoropyrimidine toxicity and genotype. The results from the test for trend and from dominant and recessive genetic models are shown. (a) Main effect of polymorphisms on fluoropyrimidine dose modification: markers of pharmacodynamics. † Adjusted for age, sex, previous chemotherapy and treatment regime using logistic regression ‡Fisher’s exact estimate used *Test for trend could not be calculated because of 0 observations in one or both phenotype groups for homozygotes in the minor allele. (b) Main effect of polymorphisms on fluoropyrimidine dose modification: markers of pharmacokinetics. † Adjusted for age, sex, previous chemotherapy and treatment regime using logistic regression ‡Fisher’s exact estimate used. (c) Main effect of polymorphisms on grade 3, 4 or 5 toxicity events: markers of pharmacodynamics. † Adjusted for age, sex, previous chemotherapy and treatment regime using logistic regression ‡Fisher’s exact estimate used. (d) Main effect of polymorphisms on grade 3, 4 or 5 toxicity events: markers of pharmacokinetics. † Adjusted for age, sex, previous chemotherapy and treatment regime using logistic regression ‡Fisher’s exact estimate used *Test for trend could not be calculated because of 0 observations in one or both phenotype groups for homozygotes in the minor allele. (DOCX) [file pone.0078053.s002.docx]

Table S4a

| **Genotype** | **No modification (%)** | | | | **Dose delay (%)** | |  | | **OR** | | **95% CI** | | **P-value** | |  | | **Adjusted OR†** | | **95% CI** | | **P-value** | |
| --- | --- | --- | --- | --- | --- | --- | --- | --- | --- | --- | --- | --- | --- | --- | --- | --- | --- | --- | --- | --- | --- | --- |
| ***TYMS,* 18p11.32 – rs45445694** | | | | | | | | | | | | | | | | | | | | | | |
| 0 | | 51 (35.4) | | | 33 (30.3) | |  | | 1.00 | |  | |  | |  | | 1.00 | |  | |  | |
| 1 | | 61 (42.4) | | | 55 (50.5) | |  | | 1.39 | | 0.79-2.47 | | 0.254 | |  | | 1.32 | | 0.72-2.40 | | 0.368 | |
| 2 | | 32 (22.2) | | | 21 (19.3) | |  | | 1.01 | | 0.50-2.05 | | 0.969 | |  | | 1.16 | | 0.55-2.47 | | 0.691 | |
| Trend | |  | | |  | |  | |  | |  | | 0.813 | |  | |  | |  | | 0.595 | |
| 1+2 vs 0 (dominant) | |  | | |  | |  | | 1.26 | | 0.74-2.16 | | 0.391 | |  | | 1.27 | | 0.72-2.23 | | 0.405 | |
| 2 vs 0+1 (recessive) | |  | | |  | |  | | 0.84 | | 0.45-1.55 | | 0.568 | |  | | 0.99 | | 0.51-1.92 | | 0.975 | |
| ***TYMS,* 18p11.32 – G>C in 3R alleles of rs45445694** | | | | | | | | | | | | | | | | | | | | | | |
| 0 | | 77 (53.5) | | | 70 (64.2) | |  | | 1.00 | |  | |  | |  | | 1.00 | |  | |  | |
| 1 | | 55 (38.2) | | | 34 (31.2) | |  | | 0.68 | | 0.40-1.17 | | 0.159 | |  | | 0.64 | | 0.36-1.14 | | 0.128 | |
| 2 | | 12 (8.3) | | | 5 (4.6) | |  | | 0.46 | | 0.15-1.38 | | 0.155 | |  | | 0.47 | | 0.15-1.50 | | 0.205 | |
| Trend | |  | | |  | |  | |  | |  | | 0.066 | |  | |  | |  | | 0.071 | |
| 1+2 vs 0 (dominant) | |  | | |  | |  | | 0.64 | | 0.38-1.07 | | 0.087 | |  | | 0.61 | | 0.36-1.06 | | 0.079 | |
| 2 vs 0+1 (recessive) | |  | | |  | |  | | 0.52 | | 0.18-1.56 | | 0.240 | |  | | 0.56 | | 0.18-1.74 | | 0.321 | |
| ***TYMS,* 18p11.32 – rs16430** | | | | | | | | | | | | | | | | | | | | | | |
| Homozygous insertion (0) | | 69 (47.9) | | | 55 (50.5) | |  | | 1.00 | |  | |  | |  | | 1.00 | |  | |  | |
| Heterozygous (1) | | 61 (42.3) | | | 42 (38.5) | |  | | 0.86 | | 0.51-1.47 | | 0.588 | |  | | 0.86 | | 0.49-1.51 | | 0.605 | |
| Homozygous deletion (2) | | 14 (9.7) | | | 12 (11.0) | |  | | 1.08 | | 0.46-2.52 | | 0.867 | |  | | 1.02 | | 0.42-2.49 | | 0.965 | |
| Trend | |  | | |  | |  | |  | |  | | 0.882 | |  | |  | |  | | 0.822 | |
| 1+2 vs 0 (dominant) | |  | | |  | |  | | 0.90 | | 0.55-1.49 | | 0.689 | |  | | 0.89 | | 0.53-1.51 | | 0.675 | |
| 2 vs 0+1 (recessive) | |  | | |  | |  | | 1.15 | | 0.51-2.60 | | 0.739 | |  | | 1.09 | | 0.46-2.57 | | 0.845 | |
| ***MTHFR,* 1p36.3 signature – any two minor alleles for rs1801133 and rs1801131** | | | | | | | | | | | | | | | | | | | | | | |
| No minor alleles | | 12 (16.7) | | 14 (25.5) | | |  | | 1.00 | |  | |  | |  | | 1.00 | |  | |  | |
| Two minor alleles | | 60 (83.3) | | 41 (74.6) | | |  | | 0.59 | | 0.24-1.41 | | 0.226 | |  | | 0.63 | | 0.24-1.63 | | 0.342 | |
| ***MTHFR,* 1p36.3 – rs1801133** | | | | | | | | | | | | | | | | | | | | | | |
| AA | | | 64 (44.4) | | | 46 (42.2) | |  | | 1.00 | |  | |  | |  | | 1.00 | |  | |  |
| AT | | | 63 (43.8) | | | 48 (44.0) | |  | | 1.06 | | 0.62-1.81 | | 0.831 | |  | | 1.06 | | 0.61-1.86 | | 0.833 |
| TT | | | 17 (11.8) | | | 15 (13.8) | |  | | 1.23 | | 0.55-2.72 | | 0.612 | |  | | 1.22 | | 0.53-2.81 | | 0.638 |
| Trend | | |  | | |  | |  | |  | |  | | 0.629 | |  | |  | |  | | 0.650 |
| TT+AT vs AA (dominant) | | |  | | |  | |  | | 1.10 | | 0.66-1.81 | | 0.722 | |  | | 1.10 | | 0.65-1.86 | | 0.734 |
| TT vs AA+AT (recessive) | | |  | | |  | |  | | 1.19 | | 0.57-2.51 | | 0.644 | |  | | 1.18 | | 0.54-2.59 | | 0.672 |
| ***MTHFR,* 1p36.3 – rs1801131** | | | | | | | | | | | | | | | | | | | | | | |
| AA | | | 58 (40.3) | | | 59 (54.1) | |  | | 1.00 | |  | |  | |  | | 1.00 | |  | |  |
| AC | | | 77 (53.5) | | | 42 (38.5) | |  | | 0.53 | | 0.32-0.91 | | 0.019 | |  | | 0.56 | | 0.32-0.97 | | 0.039 |
| CC | | | 9 (6.3) | | | 8 (7.3) | |  | | 0.87 | | 0.31-2.43 | | 0.796 | |  | | 0.97 | | 0.33-2.84 | | 0.954 |
| Trend | | |  | | |  | |  | |  | |  | | 0.101 | |  | |  | |  | | 0.186 |
| CC+AC vs AA (dominant) | | |  | | |  | |  | | 0.57 | | 0.34-0.95 | | 0.029 | |  | | 0.60 | | 0.35-1.02 | | 0.059 |
| CC vs AA+AC (recessive) | | |  | | |  | |  | | 1.19 | | 0.44-3.19 | | 0.732 | |  | | 1.32 | | 0.47-3.72 | | 0.600 |

| ***DHFR,* 5q14.1 – 19 bp intron 1 in/del** | | | | | | | | | | | | | | |
| --- | --- | --- | --- | --- | --- | --- | --- | --- | --- | --- | --- | --- | --- | --- |
| Homozygous insertion (0) | 42 (29.2) | | | 20 (18.4) |  | | 1.00 |  |  |  | 1.00 |  |  | |
| Heterozygous (1) | 72 (50.0) | | | 63 (57.8) |  | | 1.84 | 0.97-3.48 | 0.058 |  | 2.19 | 1.12-4.28 | 0.023 | |
| Homozygous deletion (2) | 30 (20.8) | | | 26 (23.9) |  | | 1.82 | 0.85-3.89 | 0.117 |  | 2.06 | 0.93-4.56 | 0.075 | |
| Trend |  | | |  |  | |  |  | 0.111 |  |  |  | 0.070 | |
| 1+2 vs 0 (dominant) |  | | |  |  | | 1.83 | 1.00-3.37 | 0.048 |  | 2.15 | 1.13-4.08 | 0.020 | |
| 2 vs 0+1 (recessive) |  | | |  |  | | 1.19 | 0.65-2.16 | 0.567 |  | 1.19 | 0.63-2.25 | 0.583 | |
| ***MTHFD1,* 14q24 – rs2236225** | | | | | | | | | | | | | | |
| GG | 46 (31.9) | | | 35 (32.1) |  | | 1.00 |  |  |  | 1.00 |  |  | |
| AG | 69 (47.9) | | | 54 (49.5) |  | | 1.03 | 0.58-1.81 | 0.922 |  | 0.98 | 0.54-1.77 | 0.941 | |
| AA | 29 (20.1) | | | 20 (18.4) |  | | 0.91 | 0.44-1.87 | 0.790 |  | 1.06 | 0.50-2.55 | 0.833 | |
| Trend |  | | |  |  | |  |  | 0.828 |  |  |  | 0.909 | |
| AA+AG vs GG (dominant) |  | | |  |  | | 0.99 | 0.58-1.69 | 0.978 |  | 1.00 | 0.57-1.75 | >0.999 | |
| AA vs GG+AG (recessive) |  | | |  |  | | 0.89 | 0.47-1.68 | 0.722 |  | 1.07 | 0.55-2.09 | 0.837 | |
| ***SHMT1,* 17p11.2 – rs1979277** | | | | | | | | | | | | | | |
| CC | | 63 (43.8) | 62 (56.9) | | |  | 1.00 |  |  |  | 1.00 |  | |  |
| CT | | 69 (47.9) | 38 (34.9) | | |  | 0.56 | 0.33-0.96 | 0.031 |  | 0.61 | 0.35-1.05 | | 0.077 |
| TT | | 12 (8.3) | 9 (8.3) | | |  | 0.76 | 0.30-1.94 | 0.569 |  | 0.97 | 0.36-2.63 | | 0.949 |
| Trend | |  |  | | |  |  |  | 0.104 |  |  |  | | 0.279 |
| TT+CT vs CC (dominant) | |  |  | | |  | 0.59 | 0.35-0.98 | 0.039 |  | 0.65 | 0.39-1.11 | | 0.113 |
| TT vs CC+CT (recessive) | |  |  | | |  | 0.99 | 0.40-2.45 | 0.983 |  | 1.22 | 0.46-3.22 | | 0.684 |

Table S4b

| **Genotype** | **No modification (%)** | | | | **Dose delay (%)** | | |  | **OR** | **95% CI** | **P-value** | |  | | **Adjusted OR†** | **95% CI** | | **P-value** | |  |
| --- | --- | --- | --- | --- | --- | --- | --- | --- | --- | --- | --- | --- | --- | --- | --- | --- | --- | --- | --- | --- |
| ***DPYD,* 1p22 signature – any minor alleles for rs3918290, 1236G>A, rs67376798 and c1129-5923C>G** | | | | | | | | | | | | | | | | | | | |  |
| All homozygous | | 140 (97.2) | | 98 (89.9) | | |  | | 1.00 |  |  | |  | | 1.00 |  | |  | |  |
| One heterozygote | | 4 (2.8) | | 11 (10.1) | | |  | | 3.93 | 1.20-12.91 | 0.015 | |  | | 3.96 | 1.17-13.33 | | 0.026 | |  |
| ***DPYD,* 1p22– rs3918290*** | | | | | | | | | | | | |  |  |  |  |  |  |  |  |
| GG | | 143 (99.3) | | 107 (98.2) | | |  | | 1.00 |  |  | |  |  |  |  |  |  |  |  |
| AG | | 1 (0.7) | | 2 (1.8) | | |  | | 2.67 | 0.14-158.7 | ‡0.579 | |  |  |  |  |  |  |  |  |
| ***DPYD,* 1p22 – 1236G>A*** | | | | | | | | | | | | |  |  |  |  |  |  |  |  |
| GG | | 141 (97.9) | | 102 (93.6) | | |  | | 1.00 |  |  | |  |  |  |  |  |  |  |  |
| CG | | 3 (2.1) | | 7 (6.4) | | |  | | 3.23 | 0.71-19.70 | ‡0.105 | |  |  |  |  |  |  |  |  |
| ***DPYD,* 1p22 – rs67376798*** | | | | | | | | | | | | |  |  |  |  |  |  |  |  |
| AA | | 144 (100) | | 107 (87.2) | | |  | |  |  |  | |  |  |  |  |  |  |  |  |
| AT | | 0 (0.0) | | 2 (1.8) | | |  | |  |  |  | |  |  |  |  |  |  |  |  |
| ***DPYD,* 1p22 – c1129-5923C>G*** | | | | | | | | | | | | |  |  |  |  |  |  |  |  |
| CC | | 141 (97.9) | | 102 (93.6) | | |  | | 1.00 |  |  | |  |  |  |  |  |  |  |  |
| CG | | 3 (2.1) | | 7 (6.4) | | |  | | 3.23 | 0.71-19.70 | ‡0.105 | |  |  |  |  |  |  |  |  |
| ***UMPS,*3q13 – rs1801019** | | | | | | | | | | | | | | | | | | | | |
| GG | | 100 (69.4) | | | | 74 (67.8) |  | | 1.00 |  |  |  | |  | | |  | |  | |
| CG | | 40 (27.8) | | | | 33 (30.3) |  | | 1.11 | 0.64-1.94 | 0.699 |  | |  | | |  | |  | |
| CC | | 4 (2.8) | | | | 2 (1.8) |  | | 0.68 | 0.06-4.87 | ‡>0.999 |  | |  | | |  | |  | |
| Trend | |  | | | |  |  | |  |  | 0.926 |  | |  | | |  | |  | |
| CC+CG vs GG (dominant) | |  | | | |  |  | | 1.07 | 0.63-1.84 | 0.792 |  | | 1.01 | | | 0.57-1.79 | | 0.966 | |
| CC vs GG+CG (recessive) | |  | | | |  |  | | 0.65 | 0.06-4.67 | ‡0.702 |  | |  | | |  | |  | |
| ***CDA,* 1p36 – rs2072671** | | | | | | | | | | | | | | | | | | | |  |
| AA | | | 67 (46.5) | | 46 (42.2) | | |  | 1.00 |  |  | |  | | 1.00 |  | |  | |  |
| AG | | | 60 (41.7) | | 50 (45.9) | | |  | 1.21 | 0.71-2.07 | 0.475 | |  | | 1.07 | 0.61-1.88 | | 0.802 | |  |
| GG | | | 17 (11.8) | | 13 (11.9) | | |  | 1.11 | 0.49-2.52 | 0.796 | |  | | 1.23 | 0.53-2.89 | | 0.632 | |  |
| Trend | | |  | |  | | |  |  |  | 0.605 | |  | |  |  | | 0.632 | |  |
| GG+AG vs AA (dominant) | | |  | |  | | |  | 1.19 | 0.72-1.97 | 0.494 | |  | | 1.11 | 0.65-1.87 | | 0.704 | |  |
| GG vs AA+AG (recessive) | | |  | |  | | |  | 1.01 | 0.47-2.19 | 0.977 | |  | | 1.19 | 0.53-2.68 | | 0.673 | |  |
| ***TYMP,* 22q13 – rs11479*** | | | | | | | | | | | | | | | | | | | |  |
| CC | | | 125 (86.8) | | 82 (75.2) | | |  | 1.00 |  |  | |  | | 1.00 |  | |  | |  |
| CT | | | 19 (13.2) | | 25 (22.9) | | |  | 2.01 | 1.03-3.90 | 0.037 | |  | | 1.83 | 0.91-3.65 | | 0.088 | |  |
| TT | | | 0 (0.0) | | 2 (1.8) | | |  |  |  |  | |  | |  |  | |  | |  |
| TT+CT vs CC (dominant) | | |  | |  | | |  | 2.17 | 1.12-4.18 | 0.018 | |  | | 2.02 | 1.03-4.00 | | 0.042 | |  |
| ***TYMP,* 22q13 – rs112723255** | | | | | | | | | | | | | | | | | | | |  |
| GG | | | 133 (92.4) | | 101 (92.7) | | |  | 1.00 |  |  | |  | |  |  | |  | |  |
| AG | | | 10 (6.9) | | 7 (6.4) | | |  | 0.92 | 0.34-2.51 | 0.873 | |  | |  |  | |  | |  |
| AA | | | 1 (0.7) | | 1 (0.9) | | |  | 1.32 | 0.02-104.13 | ‡>0.999 | |  | |  |  | |  | |  |
| Trend | | |  | |  | | |  |  |  | 0.984 | |  | |  |  | |  | |  |
| AA+AG vs GG (dominant) | | |  | |  | | |  | 0.96 | 0.37-2.47 | 0.929 | |  | | 0.95 | 0.34-2.60 | | 0.914 | |  |
| AA vs GG+AG (recessive) | | |  | |  | | |  | 1.32 | 0.02-104.6 | ‡>0.999 | |  | |  |  | |  | |  |

Table S4c

| **Genotype** | **No toxicity events (%)** | | | | | **Toxicity events (%)** | | |  | | **OR** | | **95% CI** | | **P-value** |  | | **Adjusted OR†** | | **95% CI** | | **P-value** | |  |
| --- | --- | --- | --- | --- | --- | --- | --- | --- | --- | --- | --- | --- | --- | --- | --- | --- | --- | --- | --- | --- | --- | --- | --- | --- |
| ***TYMS,* 18p11.32 – rs45445694** | | | | | | | | | | | | | | | | | | | | | | | |  |
| 0 | | | 68 (32.5) | | | 16 (36.4) | | |  | | 1.00 | |  | |  |  | | 1.00 | |  | |  | |  |
| 1 | | | 97 (46.4) | | | 19 (43.2) | | |  | | 0.83 | | 0.40-1.74 | | 0.625 |  | | 0.79 | | 0.36-1.73 | | 0.554 | |  |
| 2 | | | 44 (21.1) | | | 9 (20.5) | | |  | | 0.87 | | 0.35-2.15 | | 0.761 |  | | 1.10 | | 0.41-2.91 | | 0.855 | |  |
| Trend | | |  | | |  | | |  | |  | |  | | 0.714 |  | |  | |  | | 0.985 | |  |
| 1+2 vs 0 (dominant) | | |  | | |  | | |  | | 0.84 | | 0.43-1.67 | | 0.625 |  | | 0.87 | | 0.42-1.80 | | 0.705 | |  |
| 2 vs 0+1 (recessive) | | |  | | |  | | |  | | 0.96 | | 0.43-2.16 | | 0.930 |  | | 1.25 | | 0.52-3.02 | | 0.616 | |  |
| ***TYMS,* 18p11.32 – G>C in 3R alleles of rs45445694** | | | | | | | | | | | | | | | | | | | | | | | |  |
| 0 | | | 122 (58.4) | | | 25 (56.8) | | |  | | 1.00 | |  | |  |  | | 1.00 | |  | |  | |  |
| 1 | | | 73 (34.9) | | | 16 (36.4) | | |  | | 1.07 | | 0.53-2.14 | | 0.849 |  | | 0.90 | | 0.42-1.92 | | 0.792 | |  |
| 2 | | | 14 (6.7) | | | 3 (6.8) | | |  | | 1.05 | | 0.28-3.93 | | 0.947 |  | | 1.19 | | 0.29-4.91 | | 0.806 | |  |
| Trend | | |  | | |  | | |  | |  | |  | | 0.871 |  | |  | |  | | 0.995 | |  |
| 1+2 vs 0 (dominant) | | |  | | |  | | |  | | 1.07 | | 0.55-2.06 | | 0.850 |  | | 0.94 | | 0.46-1.93 | | 0.873 | |  |
| 2 vs 0+1 (recessive) | | |  | | |  | | |  | | 1.02 | | 0.28-3.72 | | 0.977 |  | | 1.24 | | 0.31-4.95 | | 0.758 | |  |
| ***TYMS,* 18p11.32 – rs16430** | | | | | | | | | | | | | | | | | | | | | | | |  |
| Homozygous insertion (0) | | | 103 (49.3) | | | 21 (47.7) | | |  | | 1.00 | |  | |  |  | | 1.00 | |  | |  | |  |
| Heterozygous (1) | | | 86 (41.2) | | | 17 (38.6) | | |  | | 0.97 | | 0.48-1.96 | | 0.931 |  | | 0.86 | | 0.40-1.84 | | 0.692 | |  |
| Homozygous deletion (2) | | | 20 (9.6) | | | 6 (13.6) | | |  | | 1.74 | | 0.52-4.13 | | 0.460 |  | | 1.21 | | 0.40-3.63 | | 0.735 | |  |
| Trend | | |  | | |  | | |  | |  | |  | | 0.611 |  | |  | |  | | 0.935 | |  |
| 1+2 vs 0 (dominant) | | |  | | |  | | |  | | 1.06 | | 0.55-2.04 | | 0.852 |  | | 0.93 | | 0.46-1.88 | | 0.842 | |  |
| 2 vs 0+1 (recessive) | | |  | | |  | | |  | | 1.49 | | 0.56-3.97 | | 0.420 |  | | 1.30 | | 0.45-3.69 | | 0.628 | |  |
| ***MTHFR,* 1p36.3 signature – any two minor alleles for rs1801133 and rs1801131** | | | | | | | | | | | | | | | | | | | | | | | |  |
| No minor alleles | | | 19 (18.3) | 7 (30.4) | | | |  | | | 1.00 | |  | |  |  | | 1.00 | |  | |  | |  |
| Two minor alleles | | | 85 (81.7) | 16 (69.6) | | | |  | | | 0.51 | | 0.18-1.43 | | 0.193 |  | | 0.58 | | 0.19-1.80 | | 0.350 | |  |
| ***MTHFR,* 1p36.3 – rs1801133** | | | | | | | | | | | | | | | | | | | | | | | | |
| AA | | 91 (43.5) | | | 19 (43.2) | |  | | | 1.00 | |  | |  | | |  | | 1.00 | |  | |  | |
| AT | | 91 (43.5) | | | 20 (45.5) | |  | | | 1.05 | | 0.53-2.11 | | 0.885 | | |  | | 0.96 | | 0.46-2.01 | | 0.908 | |
| TT | | 27 (12.9) | | | 5 (11.4) | |  | | | 0.89 | | 0.30-2.61 | | 0.827 | | |  | | 0.78 | | 0.25-2.43 | | 0.672 | |
| Trend | |  | | |  | |  | | |  | |  | | 0.916 | | |  | |  | |  | | 0.704 | |
| TT+AT vs AA (dominant) | |  | | |  | |  | | | 1.01 | | 0.53-1.96 | | 0.965 | | |  | | 0.92 | | 0.45-1.85 | | 0.806 | |
| TT vs AA+AT (recessive) | |  | | |  | |  | | | 0.86 | | 0.31-2.39 | | 0.778 | | |  | | 0.80 | | 0.28-2.33 | | 0.683 | |
| ***MTHFR,* 1p36.3 – rs1801131** | | | | | | | | | | | | | | | | | | | | | | | | |
| AA | | 94 (45.0) | | | 23 (52.3) | |  | | | 1.00 | |  | |  | | |  | | 1.00 | |  | |  | |
| AC | | 100 (47.9) | | | 19 (43.2) | |  | | | 0.78 | | 0.40-1.52 | | 0.459 | | |  | | 0.96 | | 0.47-1.98 | | 0.917 | |
| CC | | 15 (7.2) | | | 2 (4.6) | |  | | | 0.54 | | 0.12-2.58 | | 0.437 | | |  | | 0.74 | | 0.15-3.73 | | 0.716 | |
| Trend | |  | | |  | |  | | |  | |  | | 0.328 | | |  | |  | |  | | 0.769 | |
| CC+AC vs AA (dominant) | |  | | |  | |  | | | 0.75 | | 0.39-1.43 | | 0.379 | | |  | | 0.94 | | 0.46-1.89 | | 0.852 | |
| CC vs AA+AC (recessive) | |  | | |  | |  | | | 0.62 | | 0.14-2.81 | | 0.527 | | |  | | 0.75 | | 0.16-3.66 | | 0.727 | |
| ***DHFR,* 5q14.1 – 19 bp intron 1 in/del** | | | | | | | | | | | | | | | | | | | | | | | |  |
| Homozygous insertion (0) | | | 53 (25.4) | | | 9 (20.5) | | |  | | 1.00 | |  | |  |  | | 1.00 | |  | |  | |  |
| Heterozygous (1) | | | 110 (52.6) | | | 25 (56.8) | | |  | | 1.34 | | 0.58-3.08 | | 0.491 |  | | 1.44 | | 0.59-3.51 | | 0.420 | |  |
| Homozygous deletion (2) | | | 46 (22.0) | | | 10 (22.7) | | |  | | 1.20 | | 0.48-3.44 | | 0.623 |  | | 1.09 | | 0.37-3.18 | | 0.874 | |  |
| Trend | | |  | | |  | | |  | |  | |  | | 0.620 |  | |  | |  | | 0.863 | |  |
| 1+2 vs 0 (dominant) | | |  | | |  | | |  | | 1.32 | | 0.59-2.94 | | 0.493 |  | | 1.33 | | 0.57-3.12 | | 0.513 | |  |
| 2 vs 0+1 (recessive) | | |  | | |  | | |  | | 1.04 | | 0.48-2.27 | | 0.917 |  | | 0.84 | | 0.36-1.99 | | 0.698 | |  |
| ***MTHFD1,* 14q24 – rs2236225** | | | | | | | | | | | | | | | | | | | | | | | |  |
| GG | | | 68 (32.5) | | | 13 (29.6) | | |  | | 1.00 | |  | |  |  | | 1.00 | |  | |  | |  |
| AG | | | 98 (46.9) | | | 25 (56.8) | | |  | | 1.33 | | 0.64-2.80 | | 0.444 |  | | 1.19 | | 0.54-2.61 | | 0.664 | |  |
| AA | | | 43 (20.6) | | | 6 (13.6) | | |  | | 0.72 | | 0.26-2.08 | | 0.553 |  | | 0.88 | | 0.29-2.63 | | 0.818 | |  |
| Trend | | |  | | |  | | |  | |  | |  | | 0.736 |  | |  | |  | | 0.939 | |  |
| AA+AG vs GG (dominant) | | |  | | |  | | |  | | 1.15 | | 0.56-2.34 | | 0.700 |  | | 1.11 | | 0.52-2.35 | | 0.790 | |  |
| AA vs GG+AG (recessive) | | |  | | |  | | |  | | 0.61 | | 0.24-1.54 | | 0.291 |  | | 0.79 | | 0.30-2.10 | | 0.637 | |  |
| ***SHMT1,* 17p11.2 – rs1979277** | | | | | | | | | | | | | | | | | | | | | | | |  |
| CC | | | 96 (45.9) | 29 (65.9) | | | |  | | | 1.00 | |  | |  |  | | 1.00 | |  | |  | |  |
| CT | | | 96 (45.9) | 11 (25.0) | | | |  | | | 0.38 | | 0.18-0.81 | | 0.010 |  | | 0.40 | | 0.18-0.88 | | 0.023 | |  |
| TT | | | 17 (8.1) | 4 (9.1) | | | |  | | | 0.78 | | 0.24-2.51 | | 0.675 |  | | 0.95 | | 0.27-3.37 | | 0.933 | |  |
| Trend | | |  |  | | | |  | | |  | |  | | 0.073 |  | |  | |  | | 0.152 | |  |
| TT+CT vs CC (dominant) | | |  |  | | | |  | | | 0.44 | | 0.22-0.88 | | 0.016 |  | | 0.47 | | 0.23-0.97 | | 0.041 | |  |
| TT vs CC+CT (recessive) | | |  |  | | | |  | | | 1.13 | | 0.36-3.54 | | 0.835 |  | | 1.36 | | 0.39-4.72 | | 0.631 | |  |

Table S4d

| **Genotype** | **No toxicity events (%)** | | | | **Toxicity events (%)** | | |  | | | **OR** | | **95% CI** | | **P-value** |  | | | **Adjusted OR†** | **95% CI** | **P-value** |
| --- | --- | --- | --- | --- | --- | --- | --- | --- | --- | --- | --- | --- | --- | --- | --- | --- | --- | --- | --- | --- | --- |
| ***DPYD,* 1p22 signature – any minor alleles for rs3918290, 1236G>A, rs67376798 and c1129-5923C>G** | | | | | | | | | | | | | | | | | | | | | |
| All homozygous | | | 201 (96.2) | | 37 (84.1) | | |  | | | 1.00 | |  | |  | |  | | 1.00 |  |  |
| One heterozygote | | | 8 (3.8) | | 7 (15.9) | | |  | | | 9.47 | | 1.59-14.23 | | 0.002 | |  | | 6.76 | 1.99-22.96 | 0.002 |
| ***DPYD,* 1p22– rs3918290*** | | | | | | | | | | | | | | | | | |  |  |  |  |
| GG | | 208 (99.5) | | 42 (95.5) | | |  | | | 1.00 | |  | |  | | | |  |  |  |  |
| AG | | 1 (0.5) | | 2 (4.6) | | |  | | | 9.90 | | 0.50-587.0 | | ‡0.079 | | | |  |  |  |  |
| ***DPYD,* 1p22 – 1236G>A*** | | | | | | | | | | | | | | | | | |  |  |  |  |
| GG | | 202 (96.7) | | 41 (93.2) | | |  | | | 1.00 | |  | |  | | | |  |  |  |  |
| CG | | 7 (3.4) | | 3 (6.8) | | |  | | | 2.11 | | 0.34-9.70 | | ‡0.385 | | | |  |  |  |  |
| ***DPYD,* 1p22 – rs67376798*** | | | | | | | | | | | | | | | | | |  |  |  |  |
| AA | | 209 (100) | | 42 (95.5) | | |  | | | 1.00 | |  | |  | | | |  |  |  |  |
| AT | | 0 (0.0) | | 2 (4.6) | | |  | | |  | |  | |  | | | |  |  |  |  |
| ***DPYD,* 1p22 – c1129-5923C>G*** | | | | | | | | | | | | | | | | | |  |  |  |  |
| CC | | 202 (96.7) | | 41 (93.2) | | |  | | | 1.00 | |  | |  | | | |  |  |  |  |
| CG | | 7 (3.4) | | 3 (6.8) | | |  | | | 2.11 | | 0.34-9.70 | | ‡0.385 | | | |  |  |  |  |
| ***UMPS,*3q13 – rs1801019** | | | | | | | | | | | | | | | | | | | | | |
| GG | | | 144 (68.9) | | | 30 (68.2) | | |  | | 1.00 | |  | |  | |  | |  |  |  |
| CG | | | 61 (29.2) | | | 12 (27.3) | | |  | | 0.94 | | 0.45-1.97 | | 0.878 | |  | |  |  |  |
| CC | | | 4 (1.9) | | | 2 (4.6) | | |  | | 2.40 | | 0.21-17.52 | | ‡0.289 | |  | |  |  |  |
| Trend | | |  | | |  | | |  | |  | |  | | 0.698 | |  | |  |  |  |
| CC+CG vs GG (dominant) | | |  | | |  | | |  | | 1.03 | | 0.51-2.08 | | 0.926 | |  | | 1.03 | 0.49-2.17 | 0.948 |
| CC vs GG+CG (recessive) | | |  | | |  | | |  | | 2.44 | | 0.21-17.58 | | ‡0.280 | |  | |  |  |  |
| ***CDA,* 1p36 – rs2072671** | | | | | | | | | | | | | | | | | | | | | |
| AA | | | 95 (45.5) | | 18 (40.9) | | |  | | | 1.00 | |  | |  | |  | | 1.00 |  |  |
| AG | | | 87 (41.6) | | 23 (52.3) | | |  | | | 1.40 | | 0.70-2.77 | | 0.338 | |  | | 1.15 | 0.55-2.39 | 0.708 |
| GG | | | 27 (12.9) | | 3 (6.8) | | |  | | | 0.58 | | 0.16-2.16 | | 0.416 | |  | | 0.66 | 0.17-2.54 | 0.544 |
| Trend | | |  | |  | | |  | | |  | |  | | 0.890 | |  | |  |  | 0.805 |
| GG+AG vs AA (dominant) | | |  | |  | | |  | | | 1.20 | | 0.62-2.33 | | 0.582 | |  | | 1.04 | 0.52-2.11 | 0.904 |
| GG vs AA+AG (recessive) | | |  | |  | | |  | | | 0.49 | | 0.14-1.71 | | 0.256 | |  | | 0.62 | 0.17-2.26 | 0.465 |
| ***TYMP,* 22q13 – rs11479** | | | | | | | | | | | | | | | | | | | | | |
| CC | | | 178 (85.2) | | 29 (68.9) | | |  | | | 1.00 | |  | |  | |  | |  |  |  |
| CT | | | 30 (14.4) | | 14 (31.8) | | |  | | | 2.86 | | 1.34-6.12 | | 0.005 | |  | |  |  |  |
| TT | | | 1 (0.5) | | 1 (2.3) | | |  | | | 6.14 | | 0.08-484.38 | | ‡0.267 | |  | |  |  |  |
| Trend | | |  | |  | | |  | | |  | |  | | 0.002 | |  | |  |  |  |
| TT+CT vs CC (dominant) | | |  | |  | | |  | | | 2.97 | | 1.41-6.26 | | 0.003 | |  | | 2.70 | 1.23-5.92 | 0.013 |
| TT vs CC+CT (recessive) | | |  | |  | | |  | | | 4.84 | | 0.06-381.45 | | ‡0.318 | |  | |  |  |  |
| ***TYMP,* 22q13 – rs112723255** | | | | | | | | | | | | | | | | | | | | | |
| GG | | | 192 (91.9) | | 42 (95.5) | | |  | | | 1.00 | |  | |  | |  | |  |  |  |
| AG | | | 16 (7.7) | | 1 (2.3) | | |  | | | 0.29 | | 0.04-2.24 | | 0.203 | |  | |  |  |  |
| AA | | | 1 (0.5) | | 1 (2.3) | | |  | | | 4.57 | | 0.06-360.63 | | ‡0.332 | |  | |  |  |  |
| Trend | | |  | |  | | |  | | |  | |  | | 0.722 | |  | |  |  |  |
| AA+AG vs GG (dominant) | | |  | |  | | |  | | | 0.54 | | 0.12-2.43 | | 0.413 | |  | | 0.53 | 0.11-2.54 | 0.428 |
| AA vs GG+AG (recessive) | | |  | |  | | |  | | | 4.84 | | 0.06-381.45 | | ‡0.318 | |  | |  |  |  |
